# Multi-Field Coupled Cyclic Degradation Mechanisms of Alumina Ceramic Fiber Ropes

**DOI:** 10.3390/nano16130812

**Published:** 2026-06-30

**Authors:** Hongkai Guo, Lei Shang, Hanlei Zhai, Chunlin Wang, Zhihong Han, Jiajin Xu, Jiahui Zhou, Zhiqiang Luan, Xing Peng, Wenbo Han

**Affiliations:** 1 National Key Laboratory of Science and Technology on Advanced Composites in Special Environments, Harbin Institute of Technology, Harbin 150001, China; 2 Suzhou Research Institute, Harbin Institute of Technology, Suzhou 215104, China; 3 Shenyang Aircraft Design Institute Yangzhou Collaborative Innovation Research Institute Co., Ltd., Yangzhou 225100, China; 4 Southwest Institute of Technical Engineering, China North Industries Group Corporation, Chongqing 400050, China

**Keywords:** continuous alumina fibers, ceramic matrix composites, multi-field coupling, hygrothermal cycling, degradation mechanism, microstructural evolution

## Abstract

Continuous alumina (Al_2_O_3_) fibers are critical reinforcement materials for ceramic matrix composites (CMCs) utilized in extreme high-temperature environments. While their baseline thermal and mechanical properties are well-documented, their long-term service reliability in complex, multi-field environments—specifically coupled thermal, hygral, and atmospheric conditions—remains insufficiently quantified. This study systematically investigates the degradation mechanisms of alumina ceramic fiber ropes subjected to simulated engine exhaust atmospheres and cyclic rain exposure. By integrating macroscopic tensile testing with rigorous multi-scale microstructural characterizations (SEM, XRD, TGA, and advanced surface chemical state analyses via EDS and XPS), a comprehensive degradation model is proposed. Our findings reveal a pronounced two-stage mechanical degradation behavior: an initial catastrophic strength collapse followed by a stabilization phase. We elucidate that the initial embrittlement is governed not merely by thermal damage, but fundamentally by the hydrothermal volatilization and depletion of the surface amorphous SiO_2_ binder, which annihilates the inter-fiber cooperative load-sharing capability. Concurrently, quantitative XPS and XRD analyses strongly suggest that the internal amorphous grain-boundary films undergo rapid structural rearrangement and crystallization, effectively homogenizing the microstructure and shifting the fracture mechanics from energy-dissipative crack deflection to unhindered brittle cleavage. After the preferential depletion of the amorphous silicate phase, the exposed α-Al_2_O_3_ core dictates a stabilized mechanical response. This research provides critical theoretical frameworks and experimental evidence for the life-cycle assessment and microstructural optimization of advanced oxide ceramic fibers in next-generation aerospace applications.

## 1. Introduction

Continuous alumina (Al_2_O_3_) fibers possess a combination of superior properties: high strength, thermal stability, oxidation resistance, and corrosion resistance. These attributes establish them as advanced materials for thermal structural applications, conferring significant strategic importance. In contrast to non-oxide ceramic fibers like carbon or silicon carbide, alumina fibers demonstrate significantly superior long-term oxidation resistance under high-temperature conditions [1,2,3]. Moreover, alumina fiber offers superior mechanical properties compared to other oxide ceramic fibers, including quartz fiber [4,5,6]. These exceptional characteristics make it a key reinforcement material in continuous fiber-reinforced ceramic matrix composites (CMCs). A prime example is the alumina fiber-reinforced alumina (Al_2_O_3_/Al_2_O_3_) composite [7]. Such composites exhibit significant promise as thermal structural components in aerospace vehicles and other advanced engineering fields [8,9,10].

It should be noted that a certain proportion of SiO_2_ is often introduced into continuous oxide ceramic fibers during their fabrication and service. A significant portion of this SiO_2_ exists in an amorphous state, located at grain boundaries or within the internal glassy phase of the fibers. The incorporation of amorphous SiO_2_ facilitates precursor shaping, spinning, and densification. Furthermore, it enables grain boundary “pinning” through the formation of a glassy grain-boundary film or a distinct secondary phase [11,12,13,14]. This boundary film acts as a thermodynamic barrier, effectively suppressing the abnormal grain growth of α-Al_2_O_3_ via the Zener pinning mechanism. By restricting grain boundary mobility, the nanostructured integrity of the fiber is preserved, which is a prerequisite for maintaining high-temperature tensile strength.

Historically, the industrial-scale application of continuous alumina fibers was established in the late 20th century across North America and East Asia. Representative materials include the Alumina Continuous Fiber (ALF) series and the Nextel™ series, which have seen extensive utilization in aerospace thermal protection [15,16]. Chronologically subsequent research has expanded on fabrication processes and rigorous evaluations of these oxide fibers in complex environments [17]. Although continuous oxide ceramic fibers show great potential in thermal structural applications, their long-term environmental stability is profoundly influenced by hydrothermal degradation and amorphous phase evolution within the intergranular regions, which remain core limiting factors for aero-engine components [18]. Existing studies have highlighted the destructive impacts of complex environmental exposures on the intrinsic microstructure of advanced oxide fibers [19], indicating that the evolution of intergranular networks and interfacial phases significantly alters fracture modes [20] and directly causes the decline in macroscopic load-bearing capacity [21].

In practical service environments, materials are seldom exposed to elevated temperatures alone. They typically experience coupled interactions from multiple factors, such as humidity, atmospheric corrosion, and mechanical stress. This multi-factor coupling results in a significantly more complex degradation mechanism [15]. Elevated temperature is the primary driver of degradation, initiating microstructural evolution within the material [22,23]. For instance, in alumina fibers, temperatures exceeding 1000 °C promote a gradual phase transformation from amorphous or metastable states to stable crystalline structures [24,25]. Above 1200 °C, the formation of a mullite phase occurs, leading to a substantial reduction in fiber strength [17,26]. However, amorphous SiO_2_ is thermodynamically unstable at elevated temperatures. Influenced by factors such as temperature, dwell time, atmosphere, impurities, defects, and nucleation conditions, it can undergo devitrification to form crystalline silica phases—most commonly cristobalite, though tridymite may also appear [27,28]. The crystallization “threshold” for amorphous SiO_2_ may be relatively lowered, and cumulative thermal cycling can progressively increase the volume fraction of the crystalline phase [29].

Concurrently, within the Al_2_O_3_–SiO_2_ system, the amorphous silica can react with transitional alumina or α-Al_2_O_3_ to form aluminosilicate phases. Above approximately 1100 °C, an Al–Si reaction-driven phase evolution occurs, leading to the nucleation and growth of mullite grains at higher temperatures [30,31]. Furthermore, the stability and eventual dissipation of the sub-nanoscale SiO_2_ grain-boundary film can significantly influence grain boundary migration, defect evolution, and the retention of mechanical strength [32]. It has been reported that fiber strength can diminish to approximately 60% of its initial value at temperatures surpassing 1200 °C [33]. Exacerbating this thermal degradation, the influence of humidity and atmospheric composition cannot be overlooked. In high-temperature, oxygen-rich environments, the presence of water vapor can induce a reaction with alumina to form volatile hydrates, such as Al(OH)_3_ [34]. More critically, water vapor acts as a potent catalyst for the depolymerization of the silica network. Hydrothermal attack cleaves the siloxane bonds (Si–O–Si), generating highly volatile silicic acid (Si(OH)_4_) [35,36]. This specific volatilization mechanism not only causes macroscopic mass loss but also simultaneously triggers the disintegration of the load-bearing fiber bridging network, radically accelerating mechanical failure [37,38].

Consequently, to accurately evaluate the structural integrity of these materials, there is an urgent need to conduct coupled thermo-mechanical and environmental experiments to systematically elucidate their degradation laws under complex service conditions. Extensive research has been conducted on the performance evolution of alumina fibers and their composites under multi-field coupled environments. For instance, Armani et al. systematically examined the creep behavior of 3M’s Nextel™ 720 alumina fiber at 1100–1200 °C. Their experiments revealed that the presence of water vapor increases the fiber’s creep rate by an order of magnitude and significantly reduces its lifespan compared to dry air. Microstructural analysis confirmed that water vapor accelerates mullite phase decomposition, leading to the formation of a porous alumina layer on the fiber surface [39]. Antti et al. studied the high-temperature aging of porous aluminosilicate matrix composites reinforced with alumina fibers. Their results show that prolonged exposure at 1100 °C in air causes severe embrittlement. The failure mode shifts from a ductile fiber pull-out to a brittle, monolithic ceramic-like fracture [40]. Furthermore, van Roode et al. compared the effects of different matrices on fiber damage resistance, demonstrating that a yttrium aluminum garnet (YAG) matrix can effectively raise the high-temperature performance limit of alumina fiber composites [41].

Despite these foundational studies, there is a critical gap in the quantitative correlation between the evolution of the surface chemical state (specifically the Al/Si atomic coordination) and the macroscopic progressive fracture mechanics of pristine alumina fiber ropes under sequential thermo-hygral cycling. Therefore, this study employs advanced spectroscopic techniques, including Energy-Dispersive Spectroscopy (EDS) and X-ray Photoelectron Spectroscopy (XPS), alongside standard mechanical evaluations. By rigorously modeling the specific binding energy shifts and elemental volatilization kinetics, we systematically elucidate the intrinsic mechanism governing the two-stage performance degradation of continuous alumina fibers.

## 2. Experimental Methods

### 2.1. Experimental Materials

The specimens used in this study were alumina ceramic ropes composed of alumina, quartz, and mullite fibers. Within this composition, quartz is present in an amorphous phase and reacts with mullite, existing primarily in the form of silicates. To evaluate these materials under potential aircraft in-service conditions—specifically prolonged exposure to engine exhaust and rain—laboratory-based corrosion-fatigue simulations were sequentially conducted in these two simulated environments.

To precisely replicate fuel combustion byproducts, the simulated engine exhaust environment was maintained at a constant exposure temperature of 1000 °C. The dynamic gas mixture consisted strictly of N_2_ (77%), O_2_ (19%), CO_2_ (3.4%), CO (0.033%), and NOx (0.005%) by volume. Each individual dry-stage thermal exposure lasted for 1 h, immediately followed by a wet-stage simulated rain exposure of 15 min, which collectively constituted one complete test cycle.

**Figure 1:**
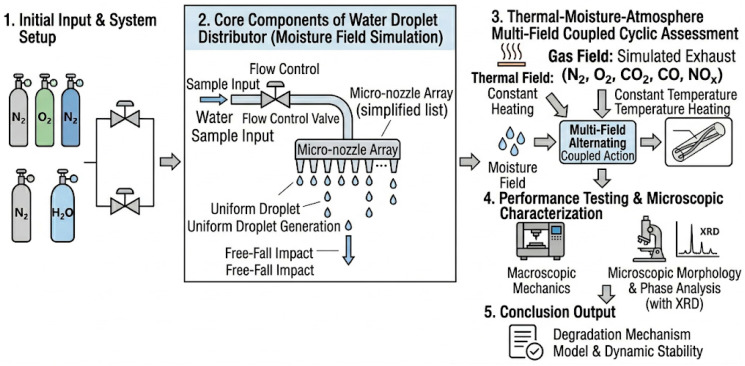
Schematic diagram of the water droplet distributor.

The second step involved a simulated rain exposure test. A custom rainfall simulation apparatus (shown in Figure 1) was constructed for this purpose. The wet-stage rain simulation was strictly conducted in accordance with the GJB 150.8A-2009 standard (Procedure III), wherein the droplet impact parameters were precisely controlled, maintaining a droplet velocity of 9 m/s and a fall height of 1 m to ensure reproducible degradation conditions. The free-fall height of the simulated rain was maintained at no less than 1 m, and the specimen was subjected to continuous exposure for a designated duration of 15 minutes. The surface area of the precipitation distributor was sufficiently large to completely encompass the upper surface of the alumina ceramic ropes, thereby guaranteeing uniform droplet impingement throughout the test. Following this exposure, the specimen was extracted and promptly advanced to the subsequent testing phase.

The multi-field coupled cyclic test protocol involves an alternating sequence of dry and wet environmental exposures. By definition, multiple consecutive dry-state exposures followed by a single wet-state exposure constitute one complete test cycle. Guided by the established performance evolution patterns of the material, specific cycle counts were selected as testing intervals to systematically conduct and record the macroscopic and microscopic performance evaluations.

### 2.2. Measurements

The microstructure of the specimens was characterized using a scanning electron microscope (SEM; ThermoFisher Apero Chemi, USA) operating at an acceleration voltage of 20 kV, a beam current of 0.4 nA, and a working distance of 10 mm. Phase composition was analyzed via X-ray diffraction (XRD; Bruker D8 Advance, Germany) with a copper target, a scan speed of 10°/min, and a scanning range from 10° to 90°. Tensile strength was measured at room temperature using an electronic universal testing machine (Instron-5569, USA) with a crosshead speed of 2 mm/min. The specimens tested were braided ropes measuring 500 mm in length and 5 mm in diameter. Stress–strain curves were recorded during testing. To guarantee reproducibility and statistical reliability, each specific cyclic condition was evaluated using at least 3 parallel specimens, with results reported as average values accompanied by standard deviations. Weight loss was determined via thermogravimetric analysis (TGA) using a simultaneous thermal analyzer (Hitachi STA200, Japan). Samples with an initial mass of approximately 10.5 mg were heated from room temperature to 1100 °C at a constant heating rate of 10 °C/min under a continuous flow of air atmosphere.

Surface chemical states and elemental compositions were quantitatively analyzed utilizing X-ray Photoelectron Spectroscopy (XPS; Thermo Scientific K-Alpha, USA) equipped with a monochromatic Al Kα source. All binding energies were calibrated with reference to the adventitious C 1s peak at 284.8 eV. Spectral deconvolution and peak fitting were systematically performed to elucidate phase transitions.

Attempts were also made to perform Raman scattering spectroscopy to assess the local structural disorder and defect states of the fibers. However, due to the severe intrinsic fluorescence background under standard laser excitation wavelengths, no resolvable Raman signals could be collected.

To guarantee the reproducibility and statistical reliability of the multi-field degradation measurements, each experimental protocol was systematically repeated. Specifically, the macroscopic mechanical evaluations (tensile tests) were conducted on at least three parallel braided rope specimens for each respective cyclic condition, with the calculated standard deviations strictly reported. Furthermore, the fundamental microstructural and thermochemical characterizations—including XRD, high-resolution XPS, and TGA—were performed in triplicate utilizing randomly extracted independent filament specimens from different batches. The high consistency of the analytical data across these independent trials robustly confirms the repeatability of the observed phase evolution and silica depletion trends.

## 3. Results and Discussion

### 3.1. Tensile Properties

**Figure 2:**
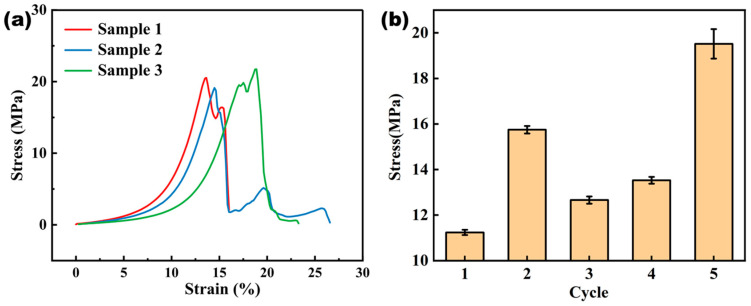
Stress–strain curves of specimens tested at room temperature: (**a**) representative parallel specimens (Samples 1–3) from the as-received batch; (**b**) variation in tensile strength of specimens after 1 to 5 cycles.

Figure 2a illustrates the typical mechanical response of a flexible fiber-based ceramic rope during tensile loading for the as-received specimen. In the initial stage, stress increases slowly and approximately linearly with strain, corresponding to the gradual straightening of the fiber bundles and the closure of internal pores and initial defects. Subsequently, the curve slope increases markedly, indicating that the load transitions from being borne by individual fibers to a cooperative state among multiple fibers. Prior to reaching the peak stress, the curve exhibits no distinct yield plateau or region of stable plastic deformation, confirming that the alumina ceramic rope does not undergo a traditional “yielding” process. After the peak stress is attained, the stress drops rapidly. In some specimens, this drop is characterized by a sharp decline followed by a slight recovery, a phenomenon typically attributed to the progressive fracture of fibers and the subsequent redistribution of load among the remaining intact fibers. Overall, the material exhibits a tensile failure mode dominated by elastic deformation with minimal plasticity before fracture, characteristic of a brittle or quasi-brittle progressive fracture process.

A comparison of Figure 2a,b reveals that hygrothermal cycling significantly affects the tensile mechanical properties of the alumina ceramic rope. Compared to the as-received specimen, the cycled specimens exhibit a substantially larger overall strain range and a notably higher strain at failure during tensile loading. This suggests that the hygrothermal environment likely weakens the constraints between fibers or at the fiber-matrix interface, allowing for greater fiber slippage and rearrangement under load. However, regarding peak stress, the curves for most cycled specimens show a reduction compared to the original, with the most pronounced decrease occurring after the initial cycles. This indicates that the hydrothermal aging effects induced by the cycling may propagate defects on the fiber surface and degrade the interfacial bonding strength, consequently reducing the overall load-bearing capacity. With an increasing number of hygrothermal cycles (from Cycle 1 to Cycle 5), the stress–strain curves exhibit a distinct evolutionary trend: specimens subjected to early cycles show a lower peak stress and a more gradual initial slope, whereas after later cycles, both the curve slope and peak stress recover slightly—though they remain below the levels of the as-received specimen. This behavior suggests that damage mechanisms dominate during the initial stages of hygrothermal cycling. After multiple cycles, however, the internal structure may gradually stabilize, and the rate of damage evolution slows.

To further elucidate the microscopic mechanisms underlying the degradation of the macroscopic tensile properties of the alumina ceramic rope subjected to hygrothermal cycling, the single-filament tensile properties of individual fibers (designated as fibers after 1–5 cyclic treatments, randomly selected from the same structural batch under varying cyclic conditions) were evaluated, with the average results summarized in Table 1.

These single-filament data strongly corroborate the macroscopic behavior of the fiber ropes previously observed. As shown in Table 1, the individual fibers exhibit distinct variations in their mechanical properties. Notably, fiber 3 demonstrates the highest single-filament tensile strength (1669.11 MPa) and the highest elongation at break (1.07%). This outstanding intrinsic strength at the microscopic scale aligns perfectly with the high-energy dissipative fracture and peak load-bearing capacity of the as-received rope specimens. Conversely, the other fiber samples exhibit varying degrees of strength reduction, dropping to 1291.83 MPa for fiber 4 and 1276.22 MPa for fiber 5, accompanied by fluctuations in their elastic modulus. This noticeable degradation at the single-filament level fundamentally explains the initial sharp decline in the macroscopic tensile strength of the ropes, which is driven by the structural damage on the fiber surface and intrinsic defects induced by the complex environments. However, as the crystalline structure gradually undergoes rearrangement and stabilization in later stages, the single-filament mechanical properties reach a new dynamic equilibrium. This microscopic evolution perfectly mirrors the macroscopic “initial decline followed by stabilization and slight recovery” trend, thereby providing compelling evidence for the underlying competition between environmental degradation and structural re-equilibration in continuous alumina fibers.

### 3.2. The Microstructure and Phase Composition of Samples

**Figure 3:**
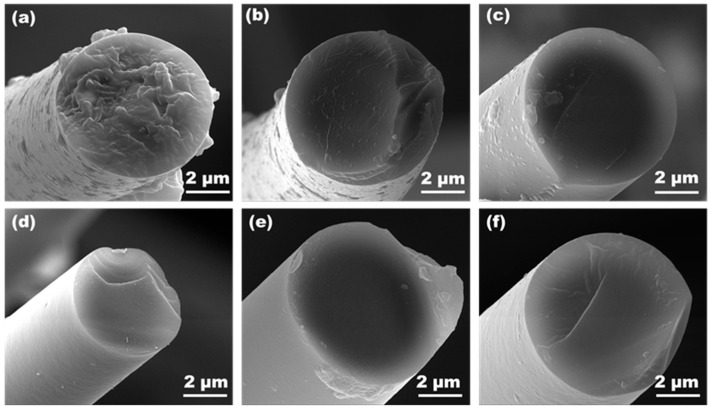
Microscopic morphology of fiber cross-sections: (**a**) as-received specimen; (**b**) specimen after 1 cycle; (**c**) specimen after 2 cycles; (**d**) specimen after 3 cycles; (**e**) specimen after 4 cycles; (**f**) specimen after 5 cycles.

The evolution of the cross-sectional microstructure with increasing cycle number was examined by SEM, as shown in Figure 3. The fracture surface of a single filament from the as-received specimen (Figure 3a) exhibits an extremely rough morphology, characterized by pronounced ridges and valleys. After five hygrothermal cycles (Figure 3f), the fracture morphology undergoes a fundamental change, transforming into a relatively flat and smooth surface with the complete disappearance of the previously complex ridges.

It should be noted that while the highly curved micro-cylindrical geometry of individual filaments precludes accurate quantitative roughness measurements via Atomic Force Microscopy (AFM), the distinct morphological contrast observed in the high-resolution SEM images rigorously validates the phenomenological transition from a particulate-bridged surface to a clean cleavage state.

To further elucidate the chemical compositional changes corresponding to this morphological shift, Energy-Dispersive Spectroscopy (EDS) analysis was performed to evaluate the elemental distribution, with the quantitative results summarized in Table 2. As shown in the table, the pristine as-received fiber exhibits a noticeable silicon (Si) content of 11.76 wt% (8.49 At%), which primarily originates from the amorphous SiO_2_ binder. Following the first hygrothermal cycle, the Si content drops to 8.93 wt%, indicating an initial partial dissolution or detachment of the surface Si-rich amorphous layer under the high-temperature and high-humidity environment. In subsequent cycles (Cycles 2 to 5), the overall mass fraction of Si recovers and stabilizes between 11.6% and 12.8%. Coupled with the morphological changes, this elemental evolution pattern suggests that while the initial cycle causes surface layer degradation, prolonged thermal exposure promotes the localized migration, redistribution, and eventual stabilization of the residual amorphous Si-containing phases rather than their complete continuous depletion. However, it must be acknowledged that EDS is a semi-quantitative technique possessing inherent measurement uncertainties, particularly for light elements such as oxygen. Therefore, the observed fluctuations in elemental concentrations must be interpreted with caution. To mitigate these inherent limitations and comprehensively verify the degradation behavior, high-resolution XPS analysis was subsequently employed as a complementary characterization. While XPS provides highly localized, surface-sensitive data, its precise quantification of specific chemical states (such as siloxane bond cleavage), when integrated with the broader elemental migration trends observed via EDS, establishes a mutually corroborative analytical framework. This synergistic combination of micro- and sub-micro-scale techniques rigorously validates the relationship between surface SiO_2_ depletion, microstructural evolution, and the definitive absence of intermediate aluminosilicate phases.

**Figure 4:**
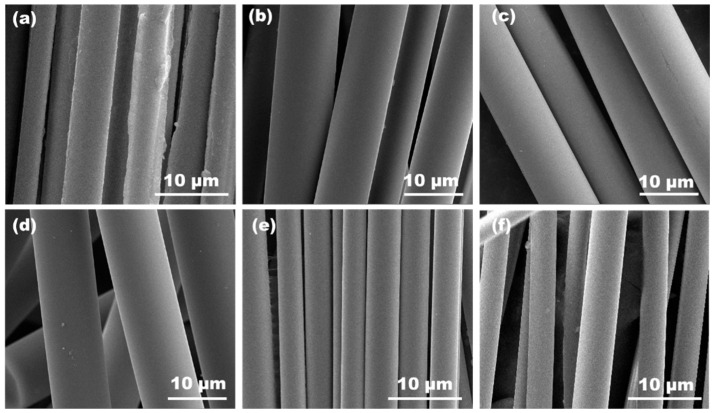
Microscopic morphology of the fiber lateral surface: (**a**) as-received specimen; (**b**) specimen after 1 cycle; (**c**) specimen after 2 cycles; (**d**) specimen after 3 cycles; (**e**) specimen after 4 cycles; (**f**) specimen after 5 cycles.

This elemental evolution and redistribution precisely corroborate the morphological changes observed on the fiber lateral surfaces, as presented in Figure 4. The surface of the fiber bundles in the as-received specimen (Figure 4a) is covered with distinct irregular particulate matter and a thin coating layer. Supported by the aforementioned EDS findings, these attachments are confirmed to be the initial Si-rich amorphous binder, which acts as a physical filler bridging the fibers. After five hygrothermal cycles (Figure 4f), the fiber surface becomes remarkably clean and smooth. This confirms that the surface amorphous phase has been structurally transformed and redistributed into inter-fiber gaps or microcracks under the coupled multi-field environment, leaving the main bodies of the individual fibers “exposed”.

**Figure 5:**
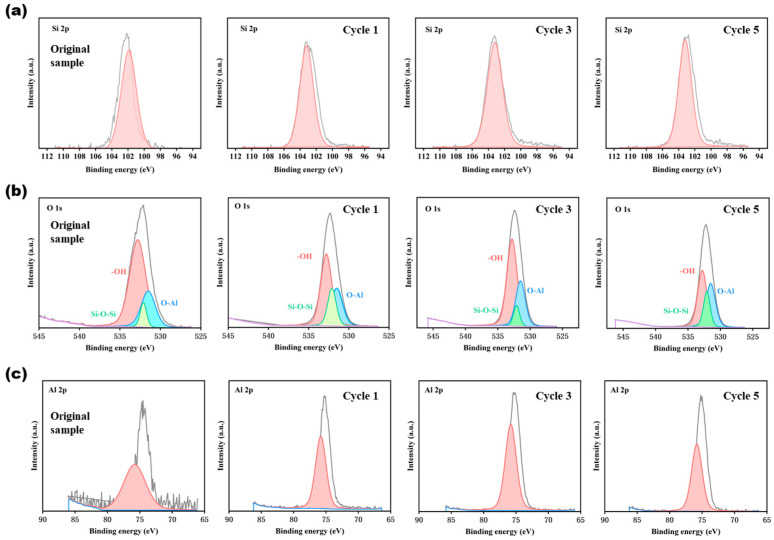
High-resolution XPS spectra of alumina fiber ropes (Cycles 0, 1, 3, and 5). (**a**) Si 2p spectra of residual silicates; (**b**) Deconvoluted O 1s spectra showing hydrothermal degradation; (**c**) Al 2p spectra verifying the absence of aluminosilicate intermediates.

To strictly quantify the relationship between silica depletion, microstructural evolution, and chemical degradation, high-resolution XPS analysis was performed across representative cycles (Figure 5).

The deconvoluted O 1s spectra (Figure 5b) provide direct evidence for the hydrothermal degradation of the inter-fiber bonding network. The spectra resolve into three distinct peaks: lattice oxygen within the α-Al_2_O_3_ core (O–Al, ~530.8 eV), hydroxyl groups (–OH, ~531.8 eV), and bridging oxygen in the silicate network (Si–O–Si, ~532.8 eV). In the pristine state (Cycle 0), the prominent Si–O–Si area fraction confirms the initial silica-rich amorphous binder. Crucially, after Cycle 1, the Si–O–Si fraction drops precipitously while the –OH signature surges. This transition quantitatively demonstrates that water vapor effectively cleaves the stabilizing siloxane network into volatile silanol species, annihilating inter-fiber physical bonding and driving the initial macroscopic strength collapse. In later stages (Cycles 3 and 5), lattice oxygen (O–Al) assumes absolute dominance, confirming that the exposed alumina core governs the stabilized mechanical response.

Concurrently, the Si 2p spectra (Figure 5a) elucidate the structural response of the residual amorphous phase. From Cycle 0 to Cycle 5, the full width at half maximum (FWHM) of the Si 2p peak exhibits a progressive narrowing. Since photoelectron line broadening in non-crystalline materials correlates directly with structural disorder, this narrowing signifies short-range structural ordering. The residual silica network transitions toward a more ordered thermodynamic state, achieving a dynamic microstructural equilibrium that aligns precisely with the late-stage mechanical stabilization.

Furthermore, the Al 2p spectra (Figure 5c) substantiate the overall phase stability of the matrix. Across all conditions, the Al 2p signal maintains an invariant, highly symmetric monomodal profile centered at ~74.5 eV. The absence of a high-binding-energy shoulder (typically >75.0 eV, characteristic of aluminosilicate tetrahedra) strongly implies that no stable, macroscopic solid-state reactions forming mullite-related intermediate structures were detected within the investigated thermal regime. This indicates that, under the current multi-field coupled testing conditions and within the detection limits of the characterization techniques, the Al_2_O_3_ matrix and the residual SiO_2_ maintain essential phase independence without forming a stabilized intermediate aluminosilicate continuous network.

**Figure 6:**
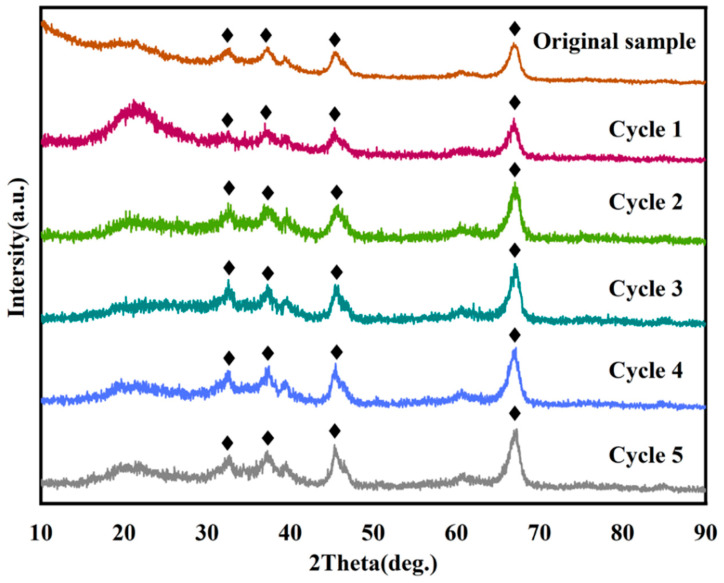
XRD patterns of the specimens.

Figure 6 presents the XRD patterns of the as-received specimen and those after 1 to 5 hygrothermal cycles. The pattern for the as-received specimen exhibits a pronounced broad, diffuse peak in the 20°–25° 2θ range, indicating a high content of amorphous SiO_2_ in the pristine material. Concurrently, diffraction peaks corresponding to the α-Al_2_O_3_ crystalline phase are present at 32.68°, 37.40°, 45.32°, and 67.28° 2θ. However, these peaks are broadened and of low intensity, suggesting poor crystallinity.

After one hygrothermal cycle, the intensity of the low-angle amorphous peak decreases significantly. The intensity ratio of the peak near 21° to the main α-Al_2_O_3_ peak at 67.28° drops from 1.26 in the as-received specimen to 0.52. As corroborated by the previous EDS analysis, this sharp attenuation of the amorphous halo is fundamentally linked to the initial volatilization and dissolution of the uncrystallized SiO_2_ surface binder. Furthermore, the hygrothermal environment induces structural rearrangement of the residual amorphous SiO_2_, promoting a gradual transition toward a crystalline state. Concurrently, the α-Al_2_O_3_ diffraction peaks become considerably sharper and more intense, reflecting enhanced crystallinity. This single cycle primarily facilitates amorphous phase rearrangement and alumina crystallization, without inducing long-range diffusion of Al and Si atoms.

Following the second cycle, the amorphous broad peak weakens further and begins to stabilize. The intensity of the α-Al_2_O_3_ peaks increases only slightly, with a decelerated growth rate. This marks the material’s entry into a “phase stabilization and grain coarsening” stage, where the crystallization of the remaining amorphous SiO_2_ nears completion and the alumina grains coarsen gradually while maintaining a stable phase structure. From the 3rd to the 5th cycle, the XRD profiles remain stable without noticeable variations. The intensity of the low-angle broad amorphous peak remains at a consistently low level, with the intensity ratio of the 21° feature to the 67.28° α-Al_2_O_3_ peak stabilizing between 0.42 and 0.45. No significant changes in the position or intensity of the α-Al_2_O_3_ peaks are observed. This indicates that no solid-state reaction occurs between Al_2_O_3_ and SiO_2_ during multiple cycles, and the phase composition of the material reaches a dynamic equilibrium.

To further evaluate the phase evolution, grain boundary crystallinity, and localized defect structures within the alumina matrix, Raman spectroscopy was systematically attempted. However, the continuous alumina fibers (ALFs) exhibited an overwhelmingly intense photoluminescence (fluorescence) background under standard laser excitation. This optical interference is intrinsically linked to the electronic and defect structures of the corundum crystal matrix. As elucidated by first-principles calculations [42], the presence of structural point defects, such as single and dimer F-type centers (oxygen vacancies) generated during the severe high-temperature multi-field coupled cycling, significantly distorts the local lattice vibrational environment and dictates complex optical behavior. This leads to a strong radiative recombination that completely masks the weak intrinsic Raman scattering peaks of the Al–O network. Consequently, high-resolution XPS and Fast Fourier Transform (FFT) patterns derived directly from HRTEM images were primarily relied upon in this study to rigorously verify the phase transitions and crystallographic ordering of the micro-components.

**Figure 7:**
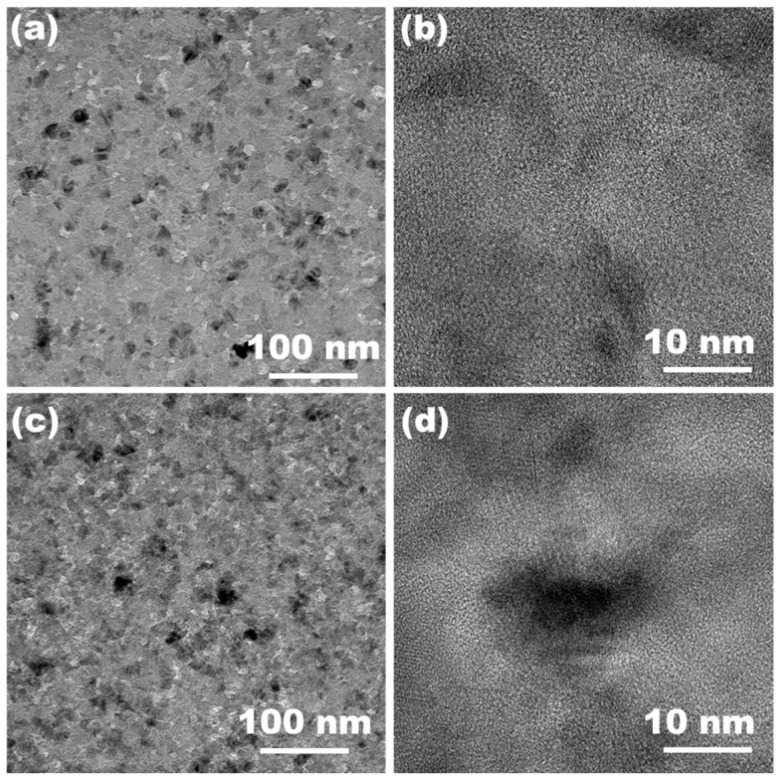
Transmission electron microscopy images: (**a**) Low-magnification TEM of C1; (**b**) High-resolution TEM of C1; (**c**) Low-magnification TEM of C5; (**d**) High-resolution TEM of C5.

Supporting evidence from transmission electron microscopy (TEM) provides detailed microstructural confirmation of the phase evolution observed in the XRD analysis. For the specimen after a single hygrothermal cycle, the low-magnification image (Figure 7a) shows a largely uniform and indistinct morphology, while the high-resolution TEM image (Figure 7b) reveals a matrix dominated by a fuzzy, chaotic amorphous structure, representative of the SiO_2_-rich phase. Within this amorphous field, only dispersed, tiny clusters with vague lattice fringes are sparsely observed, confirming the initial “poor crystallinity” and nascent crystallization from an amorphous state discussed in the XRD section.

In stark contrast, after five complete hygrothermal cycles, the microstructure exhibits significant development. At low magnification (Figure 7c), defined, darker areas with clear boundaries are evident, representing the newly formed and grown grain structure. This evolution is further detailed in the high-magnification image (Figure 7d), which displays distinct and abundant lattice fringes spanning throughout multiple well-defined crystallites. The contrast between the sparse, poorly formed nascent clusters after one cycle (Figure 7a,b) and the numerous, coarsened, well-defined grains after five cycles (Figure 7c,d) provides visual confirmation of the “grain coarsening” stage, where initial small crystallites evolve into a stable grain structure with a dynamic phase equilibrium.

Analysis of the overall trends reveals that the YHL-XWS alumina ceramic rope undergoes a two-stage microstructural evolution during hygrothermal cycling, characterized by “rapid amorphous phase crystallization followed by crystalline phase stabilization.” Supported by both XRD and TEM findings, the early stage (cycles 1–2) shows that the coupled thermal-hygral effects induce structural relaxation and rearrangement of the amorphous SiO_2_. This leads to a gradual transition toward a crystalline state via short-range atomic diffusion. Concurrently, the α-Al_2_O_3_ grains undergo coarsening and exhibit enhanced crystallinity, as evidenced by the sharpened diffraction peaks and the emergence of distinct nascent clusters, though no polymorphic transformation of alumina occurs. In the later stage (cycles 3–5), the crystallization of amorphous SiO_2_ is essentially complete, and both the grain size and crystal structure of α-Al_2_O_3_ stabilize to reach a dynamic equilibrium.

**Figure 8:**
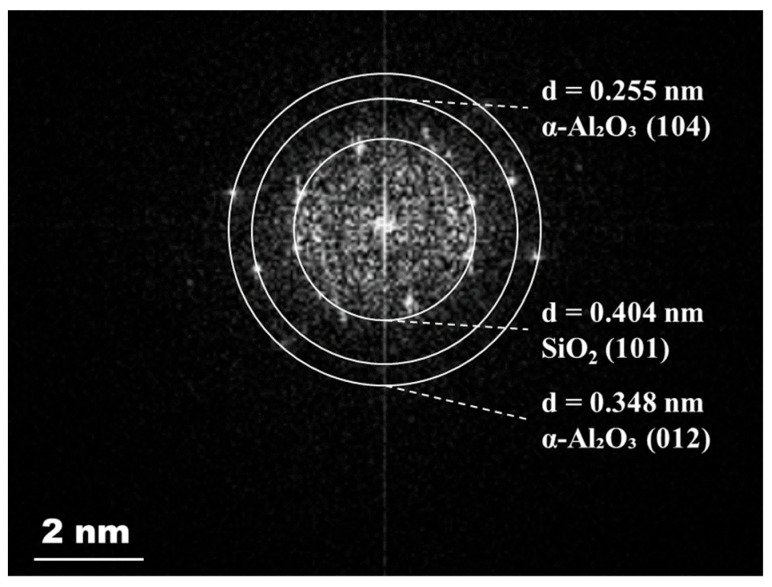
Fast Fourier Transform (FFT) pattern derived from the HRTEM image of the alumina ceramic fiber after multi-field hygrothermal cycling.

To further substantiate the crystallographic evolution and unequivocally identify the newly formed nanocrystals observed in the HRTEM analysis, Fast Fourier Transform (FFT) analysis was applied directly to the localized HRTEM images of the specimen subjected to five hygrothermal cycles. The corresponding FFT pattern (Figure 8) exhibits distinct reciprocal lattice features manifesting as concentric diffraction rings speckled with bright diffraction spots, characteristic of a well-developed polycrystalline structure. The interplanar spacings (d-spacings) derived from this FFT analysis are measured to be 0.404 nm, 0.348 nm, and 0.255 nm. These specific structural parameters can be unambiguously indexed to the (101) crystallographic plane of crystalline SiO_2_, and the (012) and (104) planes of α-Al_2_O_3_, respectively. Crucially, the extraction of the crystalline SiO_2_ (101) diffraction signature via localized FFT provides direct, high-fidelity crystallographic evidence that the initial amorphous silica surface binder has undergone complete crystallization and structural rearrangement under the prolonged multi-field coupled environment. This definitive phase identification rigorously corroborates the aforementioned XRD and HRTEM findings, confirming that the material has transitioned from a metastable, amorphous-rich state into a coarsened, highly ordered crystalline structure that eventually reaches a dynamic phase equilibrium.

This phase evolution closely corresponds to the observed trend in tensile mechanical properties—an initial sharp decline followed by a steady recovery. During the early cycles, the disruption of the initial amorphous matrix and the concurrent microstructural reconstruction weaken interfacial bonding, leading to a significant reduction in tensile strength. In the later cycles, the crystallization of amorphous SiO_2_ and the coarsening of α-Al_2_O_3_ grains enhance the intrinsic strength of the fibers. This microstructural stabilization, combined with the redistribution of the amorphous phase between fiber bundles observed by SEM, facilitates the reconstruction of the load-bearing structure. Consequently, the tensile strength gradually recovers and stabilizes.

To quantitatively evaluate the crystallization kinetics, the average crystallite size of the α-Al_2_O_3_ phase was calculated based on the prominent and relatively isolated diffraction peak located at 2θ ≈ 67.28°. The calculations for the as-received and cycled specimens were performed utilizing the Scherrer equation (Equation (1)):

(1) $$D=\frac{K\lambda }{\beta \cos\theta }$$

where D is the average grain size, K is the Scherrer constant, λ is the X-ray wavelength, β is the full width at half maximum (FWHM), and θ is the Bragg angle.

The resulting data and calculations are presented in Table 3. Correlating the grain size data with the XRD patterns, the increase in grain size from 6.23 nm in the as-received specimen to 7.85 nm after three cycles aligns with the marked weakening of the amorphous halo and the sharpening of the crystalline peaks in the XRD patterns. This indicates that the initial hygrothermal cycles provide conditions for diffusion and grain boundary migration, driving the reduction in the amorphous phase and the perfection of the crystals, which manifests as grain growth and increased crystallinity. The subsequent decrease to 6.80 nm after four cycles and stabilization at 7.20 nm after five cycles correspond to the later-stage XRD characteristics of “largely stable peak positions and a slowing rate of spectral evolution.” This confirms that after the initial rapid crystallization/rearrangement, the material enters a relatively stable phase, where subsequent changes primarily involve fine-tuning of the phase structure rather than continuous coarsening. In summary, the two-stage evolution revealed by XRD—rapid reduction in the amorphous phase and enhancement of the crystalline phase, followed by phase stabilization in the later stage—provides direct evidence for the observed trend in grain size: an initial significant increase, followed by fluctuation and eventual stabilization at the nanoscale. This demonstrates that hygrothermal cycling drives the structure of Al_2_O_3_ nanocrystallites from an initially disordered state toward a lower-energy, more stable ordered crystalline state.

However, it must be rigorously acknowledged that the crystallite sizes derived exclusively from the Scherrer approach reflect primarily the domain size contribution to peak broadening. Given that the measured full width at half maximum (FWHM, β) inherently convolutes the effects of internal microstrain and instrumental broadening factors, the derived values (fluctuating between 6.23 nm and 7.85 nm) should be interpreted strictly as relative indicators of structural ordering and phase evolution, rather than absolute dimensional metrics. Nevertheless, despite these inherent methodological uncertainties, the overarching relative trend—an initial crystallization-driven coarsening followed by a dynamic nanoscale stabilization—remains robust and is fundamentally corroborated by the direct HRTEM observations.

### 3.3. Thermal Properties of the Sample

**Figure 9:**
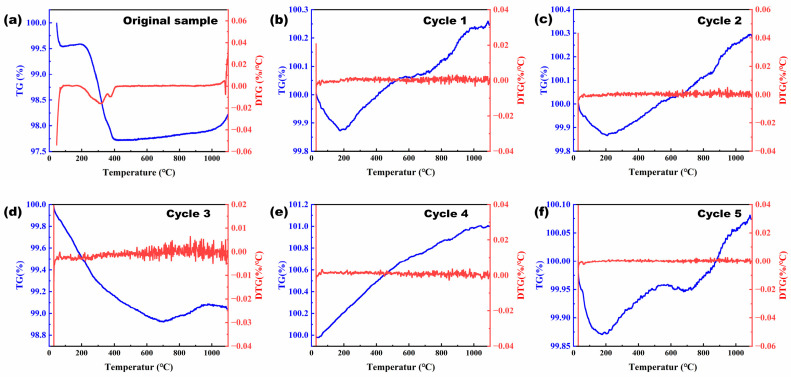
Thermogravimetric data for the specimens: (**a**) as-received specimen; (**b**) specimen after 1 cycle; (**c**) specimen after 2 cycles; (**d**) specimen after 3 cycles; (**e**) specimen after 4 cycles; (**f**) specimen after 5 cycles.

Figure 9 presents the thermogravimetric (TG/DTG) behavior of the as-received specimen and those after 1 to 5 cycles. The TG/DTG curves indicate that all specimens exhibit only minor mass changes across the entire test temperature range, demonstrating good overall thermal stability. However, some subtle differences are still observable among specimens subjected to different numbers of cycles. The as-received specimen shows a relatively distinct mass loss in the low-temperature region. As shown in Figure 9b,c, after a low number of cycles, the magnitude of this low-temperature mass loss decreases, and the TG curve becomes more gradual. This suggests that the reduction in amorphous phase content makes the interfacial regions less prone to moisture adsorption. For specimens having undergone 3 to 5 cycles, the TG curves become remarkably stable, with the DTG signal closely approaching the baseline and exhibiting only minor fluctuations. This indicates that the material has attained a state of enhanced and consistent thermal stability after multiple hygrothermal cycles.

Based on microstructural analysis, the mass change observed in the low-temperature region of the TG curves is primarily related to the amorphous phase and interfacial structure within the material. The as-received specimen, with its higher content of amorphous SiO_2_, exhibits interfacial regions that are more susceptible to moisture adsorption, resulting in a more pronounced low-temperature mass loss. After 1 to 2 cycles, XRD confirms a reduction in the amorphous phase, and SEM reveals an adjustment in the interfacial structure. This leads to a decrease in removable species, thereby diminishing the low-temperature mass loss. Following 3 to 5 cycles, both the phase composition and the interfacial structure of the material stabilize. Consequently, the low-temperature TG curves exhibit enhanced stability.

Furthermore, a slight weight gain phenomenon is observed in the high-temperature region (typically above 600–800 °C) across the TG curves. This mass increase can be attributed to the continuous oxidation of specific surface components. Given the composition of the simulated engine exhaust environment (which introduces CO_2_ alongside O_2_), it is hypothesized that partial carbonation of the surface oxides or the formation of intermediate carbon-containing species may occur during the multi-field cycling. The subsequent high-temperature TGA may calcine these localized carbonated species, contributing to the observed weight gain. However, additional direct characterization is needed to conclusively verify this speculative carbonation mechanism. Ultimately, this comprehensive thermogravimetric behavior aligns with the stabilization trend observed in the tensile properties during the later stages, further confirming that the material develops a relatively stable structural state after multiple thermal cycles.

## 4. Conclusions

This study systematically investigates the degradation mechanisms of a specific material system (YHL-XWS alumina ceramic fiber ropes) subjected to a defined set of coupled thermal, atmospheric, and hygral conditions. Based on the experimental evidence, a reasonable two-stage degradation model is proposed: an initial decline in strength followed by a gradual dynamic stabilization. While the evolution of the amorphous phase plays a central role in this proposed model, it is anticipated that the long-term degradation behavior is also fundamentally affected by defect chemistry, grain-boundary structure, and local compositional heterogeneity, features which deserve greater attention in subsequent investigations. During the initial cycling stage, the hygrothermal environment triggers severe volatilization and dissolution of the surface Si-rich amorphous SiO_2_ binder. The preferential depletion of this rigid amorphous phase destroys inter-fiber physical bonding and synergistic load-bearing capacity, causing the abrupt initial drop in macroscopic strength. As cycling progresses, the internal amorphous grain-boundary films undergo rapid crystallization and structural rearrangement, with the α-Al_2_O_3_ grain size increasing from 6.23 nm to 7.85 nm before stabilizing at the nanoscale. This crystallization-induced homogenization causes intrinsic embrittlement of individual filaments, thoroughly shifting the microscopic fracture mechanism from energy-dissipative rough fracture to unhindered smooth brittle cleavage. In the later stages, despite the minimized intrinsic strength of single filaments, the exhaustion of the rigid binder leaves the fiber surfaces clean and smooth, shifting the macroscopic load-transfer mechanism from rigid matrix bonding to extensive inter-fiber sliding. The localized migration and redistribution of residual amorphous phases facilitate interfacial structural re-equilibration; this friction-dominated synergistic load-bearing mechanism endows the macroscopic rope with exceptional strain tolerance and a stable mechanical response. Furthermore, thermogravimetric analysis (TGA) reveals that structural ordering enhances the baseline thermal stability and reduces interfacial moisture adsorption. Concurrently, CO_2_ introduced from the high-temperature simulated exhaust induces localized carbonation of surface oxides; the subsequent secondary oxidation of these carbonated intermediates during heating results in a characteristic slight weight gain in the high-temperature regime. Ultimately, after complex multi-field service, the exposed α-Al_2_O_3_ core—resulting from the preferential consumption of the amorphous silicate phase—combines with the reconstructed inter-fiber frictional interface to dominate the late-stage stable mechanical response. This research provides critical theoretical frameworks and experimental evidence for the life-cycle assessment and microstructural optimization of advanced oxide ceramic fibers in next-generation aerospace applications.

Additionally, the present multi-field coupling framework strictly bounds the degradation behavior to a specific 1000 °C thermo-hygral regime; mapping the generalized applicability of this mechanistic model necessitates future systematic evaluations across varying thermal gradients and atmospheric partial pressures.

## Figures and Tables

**Table 1:** Average single-filament tensile properties of alumina fibers 1 to 5.

Fiber Sample	Max Force (mN)	Tensile Strength (MPa)	Elastic Modulus (GPa)	Elongation at Break (%)
**Cycle 1**	112.06	1426.73	152.45	0.96
**Cycle 2**	111.51	1419.80	158.22	0.90
**Cycle 3**	131.09	1669.11	148.75	1.07
**Cycle 4**	101.46	1291.83	147.74	0.87
**Cycle 5**	100.23	1276.22	137.95	1.01

**Table 2:** Relative elemental composition of the fiber samples based on EDS analysis.

Samples	O (Wt%)	Al (Wt%)	Si (Wt%)	O (At%)	Al (At%)	Si (At%)
**Cycle 0**	48.73	39.51	11.76	61.79	29.71	8.49
**Cycle 1**	39.47	51.60	8.93	52.51	40.71	6.77
**Cycle 2**	53.67	34.72	11.61	66.37	25.45	8.18
**Cycle 3**	50.66	36.50	12.83	63.63	27.18	9.18
**Cycle 4**	51.90	35.89	12.21	64.76	26.56	8.68
**Cycle 5**	52.99	34.95	12.06	65.76	25.72	8.53

**Table 3:** Grain Size Data Table.

Sample	FWHM	Bragg Angle	Grain Size
**Original sample**	1.510	33.348°	6.23 nm
**Cycle 1**	1.428	33.336°	6.58 nm
**Cycle 2**	1.377	33.314°	6.83 nm
**Cycle 3**	1.199	33.430°	7.85 nm
**Cycle 4**	1.383	33.392°	6.80 nm
**Cycle 5**	1.306	33.429°	7.20 nm

## Data Availability

The original contributions presented in the study are included in the article, further inquiries can be directed to the corresponding authors.
